# Clinical presentation of anaplastic large-cell lymphoma in the central nervous system

**DOI:** 10.3892/mco.2013.110

**Published:** 2013-04-30

**Authors:** MASASHI NOMURA, YOSHITAKA NARITA, YASUJI MIYAKITA, MAKOTO OHNO, SHINTARO FUKUSHIMA, TAKASHI MARUYAMA, YOSHIHIRO MURAGAKI, SOICHIRO SHIBUI

**Affiliations:** 1 Department of Neurosurgery and Neuro-Oncology, National Cancer Center, Tokyo 104-0045;; 2 Division of Pathology and Clinical Laboratories, National Cancer Center, Tokyo 104-0045;; 3 Department of Neurosurgery, Tokyo Women’s Medical University, Tokyo 116-8567, Japan

**Keywords:** primary central nervous system lymphoma, anaplastic large-cell lymphoma, anaplastic lymphoma kinase, T-cell lymphoma

## Abstract

The majority of primary central nervous system (CNS) lymphomas are diffuse large B-cell lymphomas (DLBCLs) and anaplastic large-cell lymphoma (ALCL) is a type of T-cell tumor that is rare in the CNS. The aim of this study was to elucidate the clinical presentation and standard therapy of ALCLs by investigating reported cases. Additionally, a case of anaplastic lymphoma kinase (ALK)-positive ALCL in a 20-year-old man who exhibited no recurrence for >5 years following high-dose methotrexate (HD-MTX) treatment was described. Twenty-six immunocompetent patients with ALCL of the CNS that were previously reported and 1 case of ALCL of the CNS treated at our hospital were investigated. Overall survival (OS) was analyzed in relation to survival factors such as age, ALK status and the treatment regimen. The male:female ratio of the patients was 19:8. Of the 27 patients, 13 (48.1%) were ALK-positive, 9 (33.3%) were ALK-negative and the ALK status was not determined in the remaining 5 patients (18.5%). ALK-positive ALCL occurred at a younger age (median age, 17 years) and exhibited a favorable course (5-year OS, 75.0%), whereas ALK-negative ALCL presented at an older age (median age, 65 years) and resulted in fatal outcomes (5-year OS, <12.5%). Similar to the findings for systemic ALCL, ALK positivity, age <40 years and chemotherapy are associated with long-term survival for ALCL of the CNS. Chemoradiotherapy including methotrexate is recommended for ALCL and the possibility of treatment with chemotherapy alone for ALK-positive ALCL is currently under consideration.

## Introduction

Primary central nervous system (CNS) lymphoma (PCNSL) is the second most common malignant brain tumor, accounting for ∼2.9% of the primary brain tumors in Japan (1). Age, performance status (PS), lactate dehydrogenase (LDH) serum levels, cerebrospinal fluid protein concentrations and the involvement of deep structures of the brain are independent predictors of survival (2). The 2-year overall survival (OS) rate varies from 24 to 85% in proportion to the prognostic score based on the aforementioned risk factors (2). Although the majority of PCNSLs are diffuse large B-cell lymphomas (DLBCLs), primary T-cell lymphoma of the CNS accounts for only 2–8.5% of PCNSLs (3,4). However, the survival of patients with primary T-cell lymphoma is similar to that of patients with B-cell PCNSL (5).

Anaplastic large-cell lymphoma (ALCL) is an uncommon type of T-cell lymphoma first reported by Stein *et al* (6). It is characterized by large pleomorphic CD30 (Ki-1)-expressing lymphoid blasts containing horseshoe-shaped nuclei (7). Currently, the fourth edition of the WHO classification of Tumors of Haematopoietic Lymphoid Tissues, published in 2008, divides systemic ALCLs into two entities: anaplastic lymphoma kinase (ALK)-positive and ALK-negative (8). In total, 60–85% of systemic ALCLs are ALK-positive lymphomas that exhibit the characteristic t(2;5) (p23;q35) translocation that produces the ALK protein (7). This type is associated with a younger age of onset and a more favorable prognosis (9–11). Patients with ALK-positive ALCL are usually younger and have a better prognosis compared to those with ALK-negative ALCL. The 5-year OS rates are 80 and 40% in ALK-positive and -negative patients, respectively (7).

Although ALCL frequently involves lymph nodes and occasionally involves extranodal sites, such as the skin, soft tissues, bone, bone marrow, liver, lungs and gastrointestinal tract, it rarely occurs in the CNS (9,12) and ALCL of the CNS is limited to case reports. A few case reviews suggested that although ALK positivity and younger age appear to be favorable prognostic factors, this disease is generally much more aggressive compared to systemic ALCL or PCNSL (13,14). However, the precise prognosis and standard treatment have not yet been determined due to the small number of reported cases.

In this study, we reviewed previously published cases of ALCL and discussed the therapeutic management of ALCL of the CNS. We also report a case study of a patient with ALK-positive brain ALCL who underwent successful treatment with high-dose methotrexate (HD-MTX) alone and has not exhibited recurrence for >5 years.

## Materials and methods

A search was conducted using PubMed for published studies in English on cases of immunocompetent patients with ALCL of the brain. The keywords used were ‘anaplastic large-cell lymphoma’, ‘ALK’ and ‘primary central nervous system lymphoma’. Information regarding age, gender, location, ALK positivity, treatment and clinical course were collected from each study.

In addition to the 13 cases reviewed by George *et al* (13), an additional 13 reported cases (12–33) were selected and a total of 27 cases were summarized, including those of the present study (Table I). The survival time of 2 patients (cases 10 and 22) was not described, 13 patients had succumbed to the disease and 12 patients exhibited no evidence of disease at the time the studies were published.

The OS for 25 patients was calculated by the Kaplan-Meier survival method. Statistical analyses were performed using JMP software version 9 (SAS Institute Inc., Cary, NC, USA).

## Results

### Patient characteristics

Details of the 27 reported cases are provided in Table II. The male:female ratio was 19:8. The median age of the patients was 20 years (range, 4.0–82).

Of the 27 patients, 13 (48.1%) were ALK-positive, 9 (33.3%) were ALK-negative and the ALK status was not determined in the remaining 5 patients (18.5%). Twenty-four patients expressed T-cell antigens and 3 patients had null-cell tumors (cases 1, 16 and 23). There was a difference in the age distribution between the ALK-positive and -negative groups. The ALK-positive tumors occurred in patients aged <40 years. Moreover, 10 out of the 13 ALK-positive patients (76.9%) were aged ≤20 years and the median age of these 13 patients was 17 years (range, 4.0–39 years). Conversely, ALK-negative tumors occurred in older patients. Out of the 9 ALK-negative patients, 8 (88.9%) were >40 years of age and the median age of these 9 patients was 65 years (range, 22–82 years). No ALK-negative patient was <20 years of age.

A single lesion was observed in 13 (48.1%) and multiple lesions in 12 patients (44.4%), including 1 case of leptomeningeal metastasis (case 10).

The postoperative treatment was chemoradiotherapy for 14 (51.9%), chemotherapy alone for 3 (11.1%), radiotherapy alone for 6 (22.2%) and supportive care alone for 4 patients (14.8%). In total, 17 patients (63.0%) received some form of chemotherapy. The chemotherapy regimens of these 17 patients were as follows: MTX-based agents for 8 (47.1%), cyclophosphamide + doxorubicin + vincristine + prednisolone (CHOP)-based agents for 4 (23.5%), both MTX- and CHOP-based agents for 3 (17.6%), other agents for 1 (5.9%) and unknown for 1 patient (5.9%).

### Clinical courses and OS

**Figure 1:**
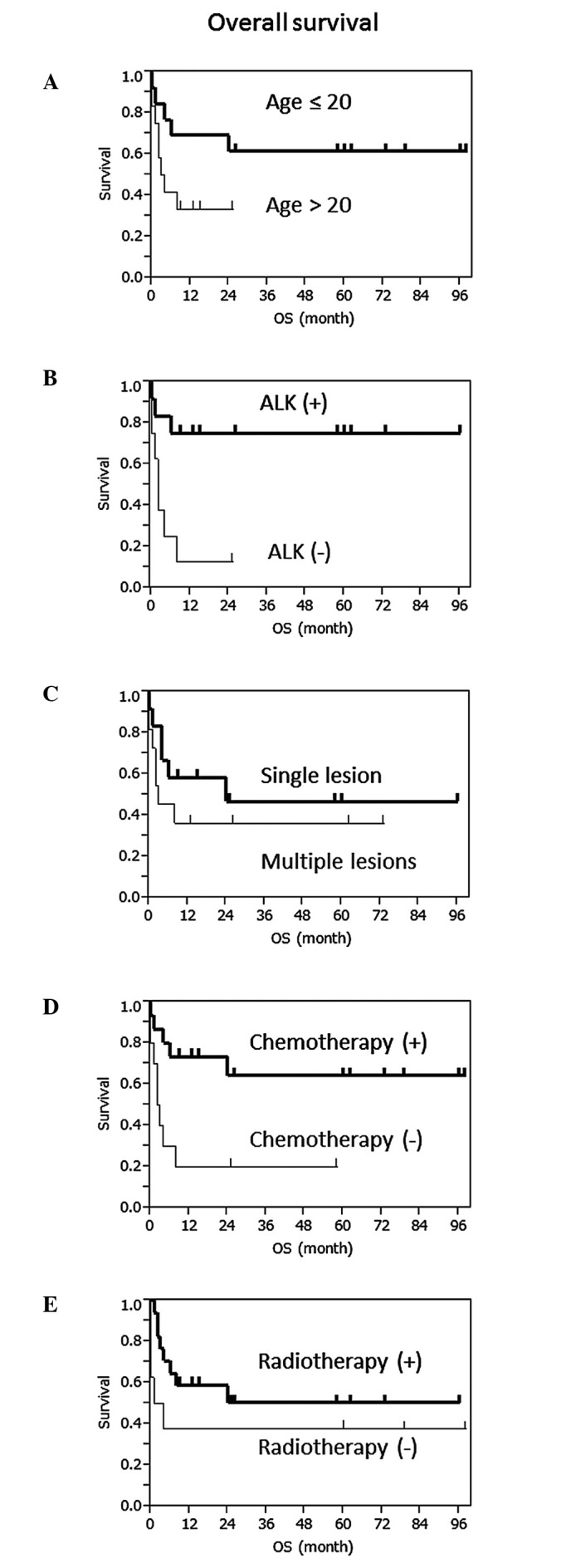
Kaplan-Meier survival curves for anaplastic large-cell lymphoma (ALCL) according to (A) age, (B) anaplastic lymphoma kinase (ALK) status, (C) number of lesions, (D) chemotherapy and (E) radiotherapy.

The patients aged <40 years (n=17) exhibited a longer OS compared to those aged >40 years (n=8) and the median OS of the groups was not reached (NR) and 2.5 months, respectively (P<0.01, log-rank test; Fig. 1A), which demonstrated younger age as a good prognostic factor. The median OS of patients aged ≤20 (n=13) and >20 (n=12) years was NR and 3.5 months, respectively (P=0.089).

In total, 10 out of the 13 ALK-positive patients (76.9%) exhibited no evidence of disease at the time of case report publication. At least 4 out of the 13 ALK-positive patients (30.7%), including our patient, exhibited no evidence of disease for >5 years after the initial diagnosis, whereas 6 out of the 9 ALK-negative patients (66.7%) succumbed to the disease within 4 months after diagnosis. The median OS of each group was NR and 2 months, respectively (P<0.01; Fig. 1B). ALK was significantly correlated with the prognosis, with a 5-year OS of 75.0 and <12.5% in patients with ALK-positive (n=12) and -negative ALCL (n=8), respectively.

There was no difference in the OS patients with a single lesion (n=12) and those with multiple lesions (n=11; P=0.36; Fig. 1C).

Chemotherapy improved patient survival, as the median OS with (n=10) or without (n=15) chemotherapy was NR and 2.5 months, respectively (P=0.01; Fig. 1D). Chemoradiotherapy tended to improve OS compared to radiotherapy alone (P=0.08); however, there was no difference in OS between patients who had received chemoradiotherapy and those who had received chemotherapy alone (P=0.73, Fig. 1E). Three patients treated with chemotherapy alone had no evidence of disease until the time when their cases were reported and 2 patients them exhibited no recurrence for >5 years.

### Case presentation

**Figure 2:**
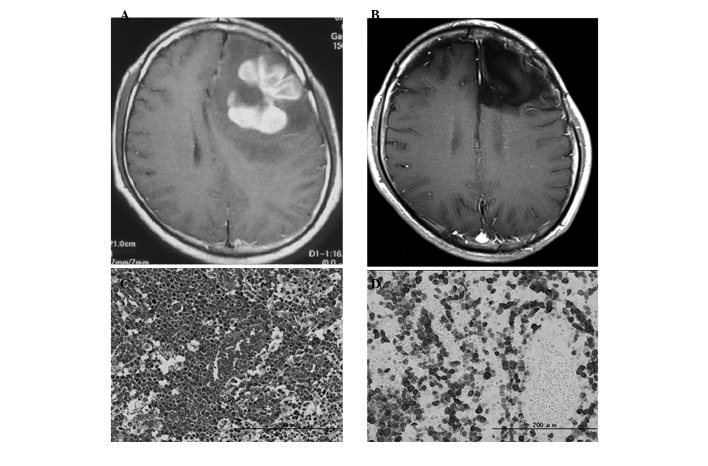
Preoperative magnetic resonance imaging (MRI) with (A) gadolinium diethylenetriamine pentaacetic acid (Gd-DTPA), (B) MRI 5 years after treatment, (C) hematoxylin and eosin (H&E) staining and (D) immunohistochemistry for anaplastic lymphoma kinase 1 (ALK-1).

We have treated 90 cases of histologically proven PCNSL from 2000 to the present at our department, including 88 cases of DLBCL and 2 cases of T-cell lymphoma, including 1 ALCL. A 20-year-old immunocompetent man with no other significant medical history was hospitalized with generalized seizures. He did not have any neurological deficits and physical examination on admission revealed no abnormal findings. Complete blood counts and serum chemistries, including LDH and soluble interleukin-2 receptor levels, were normal. Magnetic resonance imaging (MRI) with gadolinium diethylenetriamine pentaacetic acid (Gd-DTPA) revealed a well-enhanced mass lesion in the left frontal lobe (Fig. 2A), with a surrounding high-intensity lesion on T2-weighted images, indicating significant edema. Whole-body CT scans with contrast medium revealed no abnormality. Based on the suspicion of high-grade glioma, awake brain surgery and gross total removal of the tumor were performed. No residual tumor was identified on postoperative MRI with Gd-DTPA. Pathological findings revealed the proliferation of large, atypical lymphocytes containing scattered horseshoe-shaped nuclei on hematoxylin and eosin (H&E)-stained slides (Fig. 2C). Immunohistochemistry revealed that the tumor cells were positive for CD3 and ALK-1 (Fig. 2D), but negative for CD20. This patient was finally diagnosed with ALK-1-positive ALCL. Bone marrow examination findings were normal.

Treatment was immediately initiated with intravenous HD-MTX (3.5 g/m^2^). The patient completed 3 courses of HD-MTX, one every 2 weeks. Cerebral MRI and systemic positron emission tomography with fluorodeoxyglucose (FDG-PET) revealed no abnormal findings and radiotherapy was not performed. He had regular MRI examinations every 3 months until 3 years after the diagnosis and every 6 months thereafter. There has been no recurrence of the disease for 5 years (Fig. 2B) and the patient has exhibited no neurological deficits.

## Discussion

We demonstrated that ALK-positive ALCL in the CNS presents at a younger age, has a good prognosis and is sensitive to chemoradiotherapy in the analysis of reported cases.

Shenkier *et al* (5) reported a retrospective analysis of patients with T-cell PCNSL diagnosed between 1983 and 2003 at 12 institutions in 7 countries. In that report, among 25 cases of T-cell PCNSL in which the pathology report was reviewed, 3 cases (12%) exhibited the characteristics of ALCL. Thus, ALCL is a rare disease of the brain, for which the literature is limited to case reports. We collected twice as many cases as the largest reviews (13) and although our review was based on incomplete data from the literature, our findings may be of value in understanding this rare disease.

In general, ALCL of the CNS has been recognized to be significantly more aggressive compared to systemic ALCL or PCNSL (13,14). However, our data demonstrate that ALK-positive ALCL has a good prognosis, similar to systemic ALCL (10,11). Only 3 ALK-positive patients were reported to have succumbed to the disease. Tuberculosis or other infectious diseases of the CNS were suspected in these 3 patients (cases 2, 3 and 8) at the initial presentation and they were treated with antituberculosis medications or antibiotics. Delayed diagnosis may result in a worse outcome compared to that observed in other ALK-positive patients. PCNSL is rare in adolescence (34) and the radiological findings of primary ALCL in the CNS often mimic those of infectious or immunological disease. However, an early diagnosis and the timely initiation of the appropriate treatment is critical.

There is no standard treatment for primary ALCL of the CNS. Our findings demonstrate the importance of chemoradiotherapy for ALCL. Of the 17 patients treated with chemotherapy, 11 received MTX-based agents. Moreover, 9 of these 11 patients exhibited no evidence of tumor at the time of case report publication, although 2 patients died shortly after treatment. As it is known that the standard chemotherapy regimen for PCNSL is MTX and that CHOP has not been proven to be effective against PCNSL (35,36), MTX-based regimens are recommended for ALCL of the CNS. Our patient was treated with 3 courses of HD-MTX alone and he has exhibited no recurrence for >5 years. Abla *et al* (21) reported 10 pediatric cases of PCNSL, including 2 ALCLs treated with chemotherapy alone and the majority of the children achieved long-term remissions. Radiotherapy may decrease cognitive function and it is possible that ALK-positive patients, particularly younger patients, may need to start treatment with chemotherapy alone.

Our review also demonstrated that ALK-negative ALCL exhibits a poor prognosis and is very often fatal. The majority of ALK-negative patients were treated with radiotherapy or supportive care, due to their older age or poor PS. As ALK-negative ALCL tends to occur in older individuals, similar to PCNSL and DLBCL, chemoradiotherapy including HD-MTX should be initiated earlier.

In conclusion, our findings indicate that the prognosis of ALCL of the CNS is correlated with ALK positivity and patient age of <40 years. Chemoradiotherapy with MTX is recommended as the standard treatment for ALCL. However, additional multicenter studies including large numbers of cases are required.

## Figures and Tables

**Table I:** Reported cases with ALCL.

No.	ALK	Age	Gender	Location	Marker	Lesion	RT	CT regimens	Survival	Authors (Refs.)
1	+	4.5	F	Multifocal brain, brain stem, spinal cord, intra-axial and meningeal	Null-cell	M	+	CHOP	NED at 6.1 years	Havlioglu *et al* (15)
2	+	10	F	Parietal lobe abutting against falx	T-cell	S	+	CHOP, MTX	Dead at 6 months from CT	Buxton *et al* (16)
3	+	13	M	Frontal, parietal	T-cell	M	−	CHOP, MTX	Dead shortly after CT	Abdulkader *et al* (12)
4	+	29	M	Frontotemporal (macular)	T-cell	M	+	MTX	NED at 13 months	Ponzoni *et al* (17)
5	+	17	M	Parietal dura	T-cell	S	+	-	NED at 4.8 years	George *et al* (13)
6	+	18	F	Temporal dura	T-cell	M	+	CHOP, MTX	NED at 5.2 years	George *et al* (13)
7	+	9	M	Bilateral frontal	T-cell	M	+	MTX	NED at 26 months	Ozkaynak (33)
8	+	17	M	Frontoparietal eroding skull	T-cell	M	+	CHOP	Dead at 1 month	Rupani *et al* (18)
9	+	39	M	Occipitoparietal	T-cell	S	+	MTX	NED at 9 months	Cooper *et al* (19)
10	+	4	M	Pineal region, LMM	T-cell	LMM	+	CHOP	NED after CT	Karikari *et al* (20)
11	+	20	M	Region of the sylvian fissure	T cell	S	+	CHOP	NED at 8 years	Vivekanandan *et al* (22)
12	+	38	M	Parietooccipital	T-cell	S	+	MTX	NED at 15 months	Carmichael (23)
13	+	20	M	Frontal	T-cell	S	−	MTX	NED at 5 years	Present case
14	−	22	F	Dura cerebellum, temporal, 4 additional lesions	T-cell	M	−	-	Dead at 11 days	George *et al* (13)
15	−	46	F	Parietooccipital	T-cell	S	+	-	NED at 25 months	Chuang *et al* (24)
16	−	50	M	Parietal, 2 additional supratentorial, dura	Null-cell	M	+	-	Dead at 2 months	George *et al* (13)
17	−	63	M	Four frontoparietal, dura, brain	T-cell	M	+	-	Dead at 11 weeks	Paulus *et al* (25)
18	−	66	F	Temporal	T-cell	S	−	-	Dead at 4 days	Nuckols *et al* (26)
19	−	79	M	Parietoocipital	T-cell	S	−	-	Dead at 4 months	Kodama *et al* (14)
20	−	82	M	Posterior fossa lesion attaching to the tentorium	T-cell	S	−	-	Dead at 6 weeks	Gonzales (27)
21	−	75	M	Bilateral hemisphere mimicking lymphomatosis cerebri	T-cell	M	+	-	Dead at 8 months	Sugino *et al* (28)
22	−	65	M	Temporal	T-cell	S	+	MTX	NED when 2 courses of CT	Colen *et al* (29)
23	Unknown	12	F	Occipital	Null-cell	S	+	Unknown	Dead at 4 months	Bergmann and Edel (30)
24	Unknown	20	M	Parietal	T-cell	S	+	Others	Dead at 24 months	Feldges *et al* (31)
25	Unknown	63	M	Frontal, parietal	T-cell	M	+	-	Dead at 3 months	Goldbrunner *et al* (32)
26	Unknown	6	F	Unknown	T-cell	Unknown	-	MTX	NED at 79 months	Abla *et al* (34)
27	Unknown	7	M	Unknown	T-cell	Unknown	+	MTX	NED at 98 months	Abla *et al* (34)

ALCL, anaplastic large-cell lymphoma; ALK, anaplastic lymphoma kinase; RT, radiotherapy; CT, chemotherapy; M, male; F, female; M, multiple lesions; CHOP, cyclophosphamide + doxorubicin + vincristine + prednisolone; NED, no evidence of disease; S, single lesion; MTX, methotrexate; LMM, leptomeningeal metastases.

**Table II:** Summary of ALK lymphoma of reported cases.

Variable	Total	ALK-positive	ALK-negative
No.	27	13	9
Median age (range)	20.0 (4.0–82.0)	17.0 (4.0–39.0)	65.0 (22.0–82.0)
Gender (M:F)	19:8	10:3	6:3
Lesions			
Single	13	6	5
Multiple	12	7	4
Unknown	2	0	0
Initial therapy			
RT alone	6	1	4
RT+CT	14	10	1
CT alone	3	2	0
None	4	0	4
CT regimens			
MTX-based	8	5	1
CHOP-based	4	4	0
MTX+CHOP-based	3	3	0
Others	1	0	0
Unknown	1	0	0

ALK, anaplastic lymphoma kinase; M, male; F, female; RT, radiotherapy; CT, chemotherapy; CHOP, cyclophosphamide + doxorubicin + vincristine + prednisolone; MTX, methotrexate.

## Abbreviations:

CNS: central nervous system
PCNSL: primary central nervous system lymphoma
ALCL: anaplastic large-cell lymphoma
ALK: anaplastic lymphoma kinase
OS: overall survival
PS: performance status
LDH: lactate dehydrogenase
DLBCL: diffuse large B-cell lymphoma
HD-MTX: high-dose methotrexate
CHOP: cyclophosphamide + doxorubicin + vincristine + prednisolone
NR: not reached
H&E: hematoxylin and eosin
MRI: magnetic resonance imaging
FDG-PET: positron emission tomography with fluorodeoxyglucose
